# Impact of a nonnative parasitoid species on intraspecific interference and offspring sex ratio

**DOI:** 10.1038/s41598-021-02713-1

**Published:** 2021-12-01

**Authors:** Yao Zhuo Zhang, Zhengya Jin, James Rudolph Miksanek, Midori Tuda

**Affiliations:** 1 Laboratory of Insect Natural Enemies, Graduate School of Bioresource and Bioenvironmental Sciences, Kyushu University, Fukuoka, 819-0395 Japan; 2 Guangdong Key Laboratory for Innovation Development and Utilization of Forest Plant Germplasm, College of Forestry and Landscape Architecture, South China Agricultural University, Guangzhou, 510642 China; 3 Department of Entomology, University of Minnesota, Saint Paul, MN 55108 USA; 4 Laboratory of Insect Natural Enemies, Institute of Biological Control, Faculty of Agriculture, Kyushu University, Fukuoka, 819-0395 Japan

**Keywords:** Agroecology, Community ecology, Evolutionary ecology, Invasive species, Population dynamics

## Abstract

In an assemblage of multiple predators sharing a single prey species, the combined effects of the component species may scale unpredictably because of emergent interspecific interactions. Prior studies suggest that chaotic but persistent community dynamics are induced by intra-/interspecific interactions between native and nonnative parasitoids competing over a shared host. Here, we test the impact of the nonnative parasitoid *Heterospilus prosopidis* (Hymenoptera: Braconidae) on the intraspecific interference and offspring sex ratio of the native parasitoid *Anisopteromalus calandrae* (Hymenoptera: Pteromalidae). We found that the nonnative parasitoid reduced intraspecific interference among native parasitoids and decreased the proportion of female offspring produced by the native parasitoid (predicted under conditions of reduced host availability). At higher host densities, the nonnative parasitoid contributed less to the total proportion of hosts parasitized, as its innate saturating Type II response changed to a dome-shaped Type IV response with increasing density of the native parasitoid, while the native parasitoid retained its increasing Type I response. This inverse host-density-dependent response between the two parasitoids and associated competitive superiority can explain the observed changes in parasitism; at high host densities, the searching efficiency of the native parasitoid increases via host feeding while the nonnative parasitoid experiences egg limitation. These results highlight the importance of the complementary top-down effects of multiple consumers on a single resource.

## Introduction

The direct and indirect effects of predation play a crucial role in ecological communities and have wide-ranging influences on nutrient cycling, trophic organization, and eco-evolutionary dynamics^1,2^. In communities that contain multiple species of predators or parasitoids, the combined effects of each individual prey–predator (or host–parasitoid) interaction may be nonlinear rather than additive because of indirect trophic effects or the modification of direct interspecific interactions (which may be inherently nonlinear)^3^. These and other emergent properties affect the structure of multispecies assemblages and result in complex population dynamics^4–6^. To investigate the emergent properties of a multispecies assemblage, one of the simplest model systems consists of two parasitoids and a single host, in which all three engage in a number of direct and indirect interactions^7^. In this three-species system, coexisting parasitoids compete for a limited number of hosts through exploitation (host availability decreases through use^8,9^) or by interference (parasitoids directly and antagonistically interact with one another, limiting access to hosts). A moderate level of interference competition is often observed among individuals of the same species^10^. Intraspecific interference competition is referred to as “mutual interference”^11,12^.

Mutual interference reduces the searching efficiency of a parasitoid or predator as a result of antagonistic interactions among conspecifics. Hassell and Varley^11^ expressed this as

1 $$a = qP^{ - m } ,$$

where *a* is the “area of discovery” (the host or prey capture rate, or “attack rate”), *q* is the searching efficiency when conspecifics are absent, *P* is the density of parasitoids or predators, and *m* is mutual interference. The function becomes ratio dependent as *m* approaches 1. Thus, mutual interference reduces the rate at which a parasitoid interacts with its hosts by modifying the mass action term independent of host density (directly or indirectly^13–15^)—each individual parasitoid attacks fewer hosts at higher parasitoid densities, which lowers the number of parasitoid offspring per parent recruited to the next generation.

High values (close to 1) of mutual interference stabilize host–parasitoid population dynamics^11,12,16,17^ and allow for the coexistence of multiple parasitoids or predators. In natural systems, these dynamics have important implications for population ecology and community food webs^18^ (but see^19^); in an applied sense, they have been used to justify the release of multiple natural enemies in biological control programs^11,20^. In a theoretical three-species system with two consumers that share a single resource, coexistence requires trade-offs between exploitative and interference competition^21^. Asymmetric interspecific interactions are likely to influence mutual interference as well. In one such system, the parasitoids *Eupelmus vuilleti* (Hymenoptera: Eupelmidae) and *Dinarmus basalis* (Hymenoptera: Pteromalidae) exhibit different behavioral responses to interspecific competition over their shared host, the cowpea bean beetle *Callosobruchus maculatus* (Coleoptera: Chrysomelidae: Bruchinae); the more aggressive competitor, *D. basalis*, defends its hosts and (self-)superparasitizes whereas *E. vuilleti* multiparasitizes undefended hosts^22^. In another example involving four species of predaceous stoneflies (Plecoptera: Perlidae and Perlodidae) competing for blue-winged olive mayfly larvae (Ephemeroptera: Baetidae), the presence of a second predator decreased mutual interference among individuals of the first predator species^23^. As a final example, mutual interference among female *Anisopteromalus calandrae* (Hymenoptera: Pteromalidae) (a native parasitoid) was estimated to have decreased in the presence of a nonnative parasitoid, *Heterospilus prosopidis* (Hymenoptera: Braconidae), sharing a host: the azuki bean beetle *Callosobruchus chinensis* (Coleoptera: Chrysomelidae: Bruchinae); this nonnative parasitoid altered the behavior of the native parasitoid and destabilized the system^6,24^.

The degree of mutual interference among conspecific parasitoids can be estimated through individual-level observations, population-level experiments, or population models (references in^24^). For the multispecies assemblage consisting of the azuki bean beetle and two of its parasitoids (described above), the effects of the nonnative parasitoid on mutual interference among native parasitoids (and the emergent asymmetry) have been estimated based on laboratory-parameterized population models of assemblage dynamics^6^ and later supported by a study on individual behavior^24^. Additionally, the individual-level study detected a host-density-dependent change in the patch residence time and proportion parasitism by the native parasitoid in the presence of the nonnative parasitoid^24^ that was not tested at the assemblage level^6^.

If the competitive relationship between the two parasitoids were asymmetric, then the offspring sex ratio might also be affected asymmetrically. Sex allocation theory predicts that mothers in a favorable environment invest more in the offspring sex that will benefit the most from that environment^25,26^. As hosts are depleted by the nonnative parasitoid, the population density of the native parasitoid per host increases—this could lead to a decrease in the proportion of female offspring produced by the native parasitoid, if sex allocation theory applies (female *A. calandrae* benefit more from an increase in host density because they can feed directly on host fluids, termed “host feeding”, to increase their fecundity). (Alternatively, in the absence of the effects of host depletion^27^, the proportion of female offspring may also increase—when in competition, the parasitization preference of *A. calandrae* shifts toward pupae, whereas that of the nonnative parasitoid shifts toward fourth instar larvae (niche shift^28^), leaving a greater proportion of suitable hosts (pupae) for *A. calandrae*.)

The purpose of the present study is to experimentally test the following hypotheses related to the introduction of the nonnative parasitoid *H. prosopidis*: the nonnative parasitoid (1) reduces mutual interference among native parasitoids independent of host density (predicted at the assemblage^6^ and individual^24^ levels), (2) decreases the proportion of female offspring produced by the native parasitoid (in response to host depletion by the nonnative parasitoid, predicted by sex allocation theory^25,26^), (3) increases the total proportion of parasitized hosts, and (4) is unaffected by the native parasitoid in its population response to host density (predicted at the assemblage level^6^). Finally, we will discuss our results within the context of varying scales of organization, from individuals to communities.

## Materials and methods

### Study system

Our experimental system consists of one host and two parasitoids: the azuki bean beetle *Callosobruchus chinensis* and its parasitoids *Anisopteromalus calandrae* and *Heterospilus prosopidis*. This assemblage is an important model system in ecological studies^28–34^. The azuki bean beetle is a cosmopolitan pest of stored legumes (Fabaceae) and is native to East Asia^35,36^. Females lay eggs on the seed coat; after the larvae burrow through the seed coat and into the bean (which is apparent as the color of the egg changes from transparent white to pure white), they feed on the internal tissues (cotyledons) to develop through four larval instars; pupation occurs within the bean before the adults emerge. Under standard laboratory conditions (30 °C, 60% RH), development from egg to adult takes about 22 days^37^.

The parasitoids *A. calandrae* and *H. prosopidis* are used in the biological control of bruchine beetles^38^ and attack the late larval instar and pupal stages of the azuki bean beetle^6^; when the two species are in competition, the former prefers pupae and the latter prefers fourth instar larvae^28^. *A. calandrae* is a natural enemy of various stored product pests, including bean beetles (in its native range), whereas *H. prosopidis* is a nonnative control agent of bruchine beetles imported from the southern United States. Both are solitary idiobiont ectoparasitoids (solitary parasitoids usually lay one egg per host and do not readily engage in superparasitism; idiobionts prevent further development of their hosts following parasitization; ectoparasitoids feed externally on their hosts; superparasitism is to parasitize a host that was already parasitized by a conspecific). While *A. calandrae* (the native parasitoid) is synovigenic—females continue to mature eggs throughout their lives—and engages in concurrent, non-destructive host feeding to obtain nutrients for egg maturation, the nonnative parasitoid *H. prosopidis* is pro-ovigenic (or weakly synovigenic) and does not host feed (pro-ovigenic parasitoids emerge as adults with a full complement of mature eggs)^39,40^. The searching efficiency of *H. prosopidis* does not decrease except for at extremely high densities of conspecifics (pseudo-interference)^14,41^. Under standard laboratory conditions, *A. calandrae* and *H. prosopidis* reach adulthood in 12–13 days; as adults, both live for roughly five days. Females of both parasitoid species can lay up to about 40 eggs, although this varies greatly for *A. calandrae* depending on the availability of hosts for host feeding [Phyu Phyu San and Tuda, unpublished data]. For both *A. calandrae* and *H. prosopidis*, a single male can inseminate at least 10 females [^42^; MT, personal observation]. Both species have a haplodiploid sex determination system (fertilized eggs develop into females, unfertilized eggs develop into males), and a maternal female can manipulate her offspring’s sex by releasing stored sperm (received from her mate) to fertilize her eggs.

### Preparation

Our experiment tested the effects of host density and the presence or absence of the nonnative parasitoid *H. prosopidis* on mutual interference among native *A. calandrae* females. We prepared three different host densities (2, 4, or 8 larvae per bean, which represents low to high host densities) by placing 10–60 mated female beetles in Petri dishes (9 cm in diameter, 1.5 cm in height) with one layer of dried azuki beans (Fabaceae, *Vigna angularis* cv. *akadaiya*) (purchased from Daiwa-zakkoku, Hokkaido, Japan). The adults were given 24 h to oviposit before they were removed; one week later, we collected the beans with 2, 4, or 8 hatched larvae estimated by counting the number of hatched eggs. (The number and developmental stages of larvae cannot be visually inspected from outside of a bean. Once infested beans are cut open for inspection, the exposed larvae will die. However, there is a high probability that larvae survive to adulthood if reared at densities of up to about 8 larvae per bean^33^. Therefore, the number of hatched eggs is a good estimate of the number of late instar larvae or pupae.) The developing hosts were left for a total of 15 days so they could reach the fourth larval instar before being used in the experiment. Our strain of azuki bean beetles originated from Japan (strain jC).

Parasitoids were prepared by transferring 20 females and 6 males of each species from laboratory subculture into separate Petri dishes (10 females and 3 males each) that contained a layer of azuki beans with late fourth instar host larvae. The resulting offspring were allowed to develop; once the subsequent generation emerged as adults, females and males of each species were collected in separate Petri dishes (approximately 50 females and 20 males per Petri dish) and permitted to mate freely for 24 h before being used in the experiment. Our strain of *A. calandrae* originated from Japan, and *H. prosopidis* originated from Hawaii. All three insect species have been reared under laboratory conditions for more than 20 years.

### Experiment on parasitism and offspring sex ratio

We placed nine beans with one of the three different host densities (2, 4, or 8 hosts per bean) of 15-day-old larvae into a Petri dish before adding 1, 4, or 16 *A. calandrae* females (*A. calandrae* density) and 0 or 10 (absence/presence) *H. prosopidis* females. The parasitoids were removed after four days of exposure to the hosts, and the parasitoid offspring and any unparasitized hosts were allowed an additional 17 days to complete their development. The larvae, if not parasitized, developed into pupae during the 4-day exposure to the parasitoids. The numbers of female and male offspring of each parasitoid species were recorded. Each combination of host and parasitoid densities was replicated 12 times for a total of 216 experimental runs. The entire preparation and experiment were conducted in a growth chamber at 30 °C, 60% RH, and 16L:8D; parasitoids were not provided with supplemental nutrition or water when in culture, preparation, or the experiment. All methods were performed in accordance with the relevant guidelines and regulations.

### Statistical analysis

The effects of host density, *A. calandrae* density, the presence/absence of *H. prosopidis* (a categorical variable), and all interactions on the daily per capita reproduction of *A. calandrae* [log(number of offspring + 0.5)^43^] were tested using a general linear model. In this model, a significant interaction between *A. calandrae* density and the presence of *H. prosopidis* would indicate an effect of *H. prosopidis* on mutual interference among conspecific *A. calandrae*. A significant interaction between host density, *A. calandrae* density, and the presence of *H. prosopidis* would indicate a host-density-dependent effect of the nonnative parasitoid on mutual interference among *A. calandrae*. Following Eq. (1), Hassell and Varley^11^, and Shimada^41^, mutual interference *m* was independently calculated for *A. calandrae* as the slope of the individual 24-h attack rate (daily per capita reproduction, per host) as a function of conspecific parasitoid density (both log-transformed).

To investigate the proportion of hosts parasitized, a logistic regression was used to test the effects of host density, *A. calandrae* density, the presence of *H. prosopidis*, and all interactions on the number of attacked or escaped hosts. The effects of host density, *A. calandrae* density, and the presence of *H. prosopidis* on the offspring sex ratio of *A. calandrae* were also analyzed with a logistic regression model. In this model, a significant effect of *A. calandrae* density would indicate local mate competition (when the density of female parental parasitoids increases, the proportion of male offspring increases because male offspring are more likely to compete with unrelated males for mates and less likely to compete with their own brothers)^44–46^, while a significant effect of the presence of *H. prosopidis* would suggest that *A. calandrae* responds to a decrease in host availability.

Host density and *A. calandrae* density were log-transformed in all above models. All statistical tests were performed in JMP 14.2.0. Figures were prepared with R version 4.0.3.

### Modeling parasitism

A variation of the familiar Nicholson–Bailey model was used to investigate the population-level functional responses of *A. calandrae* and *H. prosopidis*. In the model, the numbers of parasitized hosts were formulated as

2 $${P}_{ Ac,t+1}={H}_{0}(1-\mathrm{exp}(-f))\mathrm{exp}\left(-g\right)$$

3 $${P}_{Hp,t+1}={H}_{0}\left(1-\mathrm{exp}\left(-g\right)\right)\mathrm{exp}\left(-f\right)$$

where ${H}_{0}$ is the initial number of hosts, ${P}_{ Ac,t+1}$ is the number of offspring produced by the native parasitoid *A. calandrae* at generation *t* + 1, ${P}_{Hp,t+1}$ is the number of offspring produced by the nonnative parasitoid *H. prosopidis* at generation *t* + 1, *f* is the response of the native parasitoid, and *g* is the response of the nonnative parasitoid. Exp(− ·), the 0th term of the Poisson distribution, indicates the proportion of hosts escaping parasitism with ·, the mean number of encounters per host^47^. The mean encounters per host for the native parasitoid *f* (Type I functional response with mutual interference^6^) and the nonnative parasitoid *g* (Type II functional response^29^) were defined as

4 $$f= {a}_{Ac}{H}_{0}{{P}_{Ac,t}}^{1-m/(1+{c}_{m}{P}_{Hp,t})}$$

5 $$g={a}_{Hp}{P}_{Hp,t}/(1+{a}_{Hp}{t}_{h}{H}_{0})$$

where ${P}_{ Ac,t}$ is the number of native female parasitoids at generation *t*, ${P}_{Hp,t}$ is the number of nonnative female parasitoids at generation *t*, ${a}_{Ac}$ is the searching efficiency of the native parasitoid (which is increased by nondestructively feeding on hosts ${H}_{0}$), ${a}_{Hp}$ is the searching efficiency of the nonnative parasitoid, *m* is the degree of mutual interference among native parasitoids, ${c}_{m}$ is a parameter for the reduction of *m* by the nonnative parasitoid, and ${t}_{h}$ is the handling time (or the inverse of egg limitation) for the nonnative parasitoid.

Based on Eqs. (2) and (3), the difference between the proportions of hosts parasitized by each parasitoid is

6 $${{(P}_{Hp,t+1}-P}_{ Ac,t+1})/{H}_{0}=\mathrm{exp}\left(-f\right)-\mathrm{exp}(-g)$$

Equation (6) was fit to the proportion parasitism data from the experiment (the observed proportion of hosts parasitized by the nonnative parasitoid subtracted by the observed proportion of hosts parasitized by the native parasitoid). Corrected AIC (AICc) scores were used to compare models for model selection. The Gauss–Newton method was used to minimize the sum of squared errors for the nonlinear model fit in JMP 14.2.0.

## Results

### Reproduction and mutual interference of *A. calandrae*

**Figure 1:**
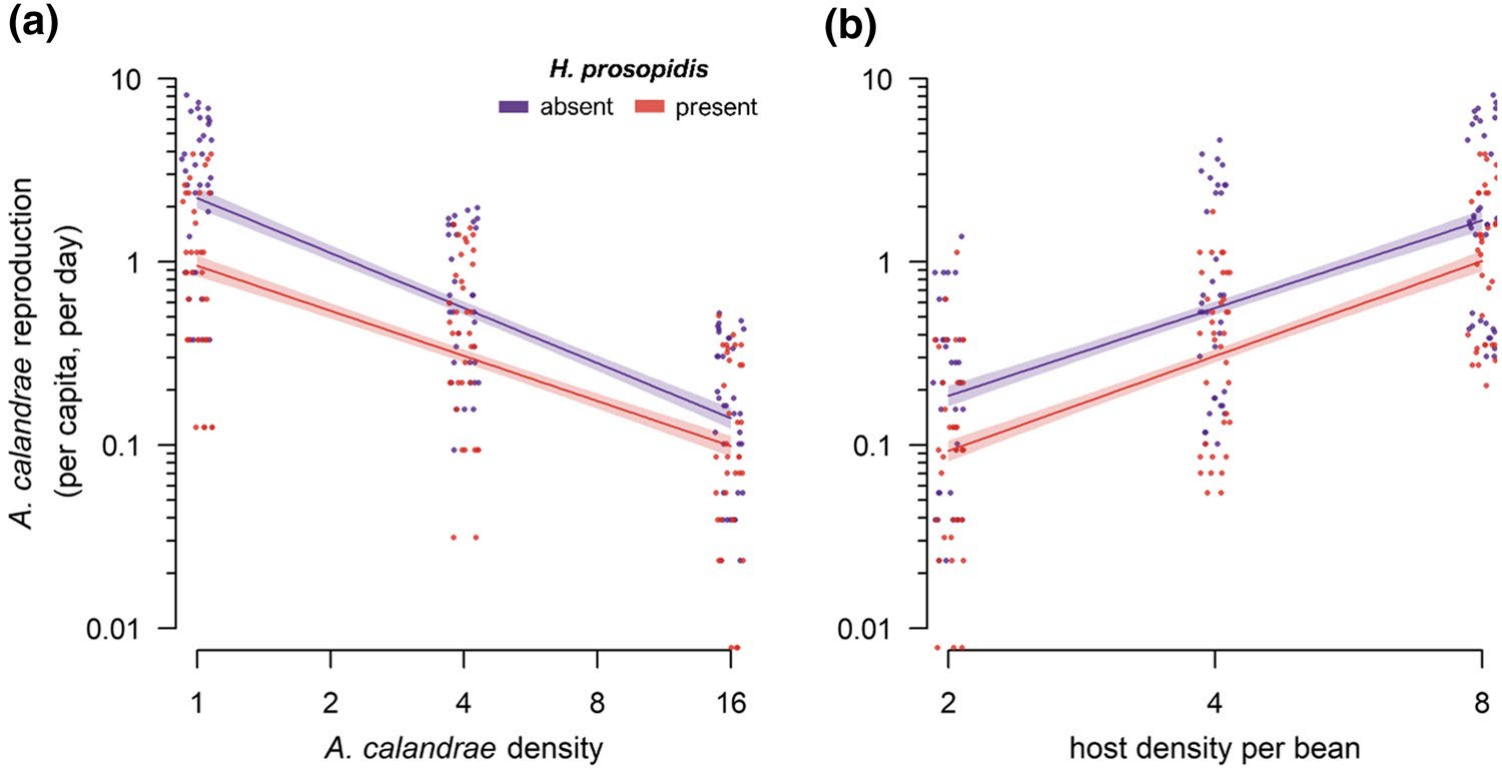
The number of emerged offspring of the native parasitoid *Anisopteromalus calandrae* (per female, per day) in the presence (red) and absence (purple) of the nonnative parasitoid *Heterospilus prosopidis* as a function of (**a**) *A. calandrae* density and the presence of *H. prosopidis* (*p* < 0.001 for the interaction between *A. calandrae* density and presence of *H. prosopidis*) and (**b**) host density (*p* < 0.001). Lines depict the general linear model fit (with shaded 95% CI). A small positive or negative random number (jitter) was added to the *x-*coordinate of each data point for graphical clarity.

In testing the effects of host density, *A. calandrae* density, the presence or absence of the nonnative parasitoid *H. prosopidis*, and all interactions on the daily per capita reproduction of *A. calandrae*, the model fit the data well (*R*^2^ = 0.918; *F*_7, 208_ = 333.3, *p* < 0.001). The daily per capita reproduction of *A. calandrae* was significantly reduced with decreasing host density (which limited host feeding), increasing *A. calandrae* density (via mutual interference), and in the presence of *H. prosopidis* (Table 1, Fig. 1). There was a significant interaction between *A. calandrae* density and the presence of *H. prosopidis* (Table 1), which indicates a negative effect of the nonnative parasitoid on mutual interference among *A. calandrae* (the slope against *A. calandrae* density was less steep when *H. prosopidis* was present; Fig. 1). While the magnitude of this effect tended to increase with host density, the three-way interaction was not statistically significant (Table 1). Mutual interference (*m*) decreased from 0.998 ± 0.040 (estimate ± SE) to 0.817 ± 0.056 in the presence of the nonnative parasitoid. There were no significant interactions between host density and *A. calandrae* density or between host density and the presence of *H. prosopidis* (Table 1).

**Table 1:** Effect tests for the general linear model of the reproduction of the native parasitoid *Anisopteromalus calandrae* (per female, per day; log-transformed) as a function of host density, *A. calandrae* density, the presence/absence of the nonnative parasitoid *Heterospilus prosopidis*, and all interactions.

Independent variable	*F* ratio	*df*	*p*
Log (host density)	1006.0	1	< 0.001
Log (*A. calandrae* density)	1209.2	1	< 0.001
Presence of *H. prosopidis*	103.4	1	< 0.001
Log (host density) × log (*A. calandrae* density)	0.3	1	0.565
Log (host density) × presence of *H. prosopidis*	1.6	1	0.214
Log (*A. calandrae* density) × presence of *H. prosopidis*	12.0	1	< 0.001
Log (host density) × log(*A. calandrae* density﻿) × presence of *H. prosopidis*	0.6	1	0.430

### Offspring sex ratio

**Figure 2:**
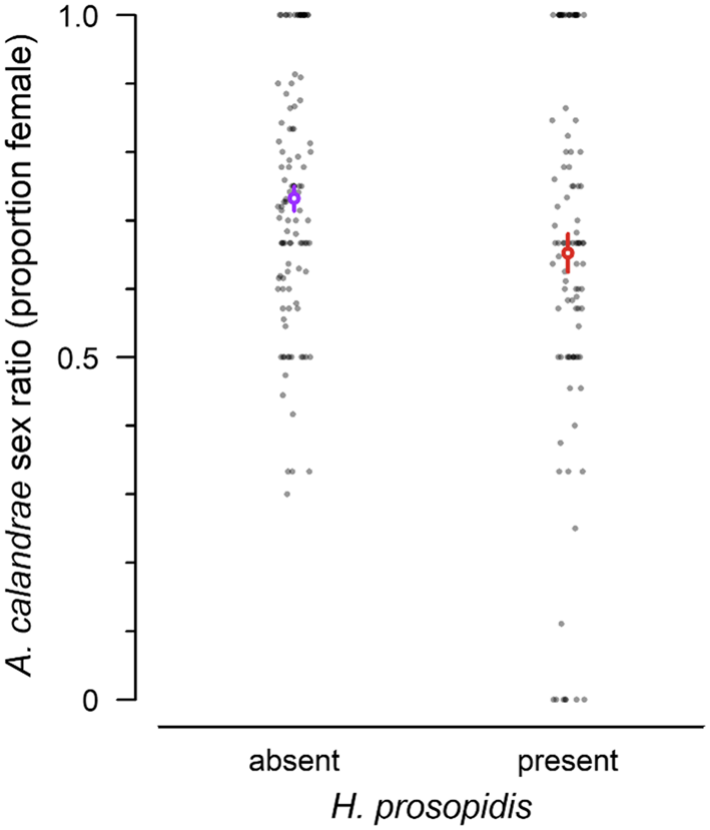
Offspring sex ratio of the native parasitoid *Anisopteromalus calandrae* in the absence and presence of the nonnative parasitoid *Heterospilus prosopidis* (*p* = 0.028). Open circles with error bars overlayed on the data indicate the mean ± SE.

Because the model fit to the sex ratio of *A. calandrae* was not improved by a three-way interaction (*χ*^2^ = 1.8, *df* = 1, *p* > 0.1), this term was removed from the model. There was a significant decrease in the offspring female ratio of *A. calandrae* in the presence of *H. prosopidis* (Table 2, Fig. 2). The effect of host density, *A. calandrae* density, and all interactions were nonsignificant (Table 2). The offspring sex ratio of *H. prosopidis* was not affected by host density, *A. calandrae* density, or the interaction between host density and *A. calandrae* density (Table 2).

**Table 2:** Effect tests for the logistic regression model of offspring sex ratio (proportion female) as a function of host density, the density of the native parasitoid *Anisopteromalus calandrae*, the presence/absence of the nonnative parasitoid *Heterospilus prosopidis*, and all two-way interactions.

Dependent variable	Independent variable	Likelihood ratio *χ*^2﻿﻿^	*df*	*p*
Sex ratio of *A. calandrae*	Log (host density)	2.52	1	0.112
	Log (*A. calandrae* density)	1.62	1	0.203
	Presence of *H. prosopidis*	4.85	1	0.028
	Log (host density) × log (*A. calandrae* density)	0.08	1	0.777
	Log (host density) × presence of *H. prosopidis*	0.27	1	0.603
	Log (*A. calandrae* density) × presence of *H. prosopidis*	1.42	1	0.233
Sex ratio of *H. prosopidis*	Log (host density)	1.72	1	0.190
	Log (*A. calandrae* density)	0.60	1	0.437
	Log (host density) × log (*A. calandrae* density)	3.02	1	0.082

### Proportion of hosts parasitized and population (functional) responses to host density

**Figure 3:**
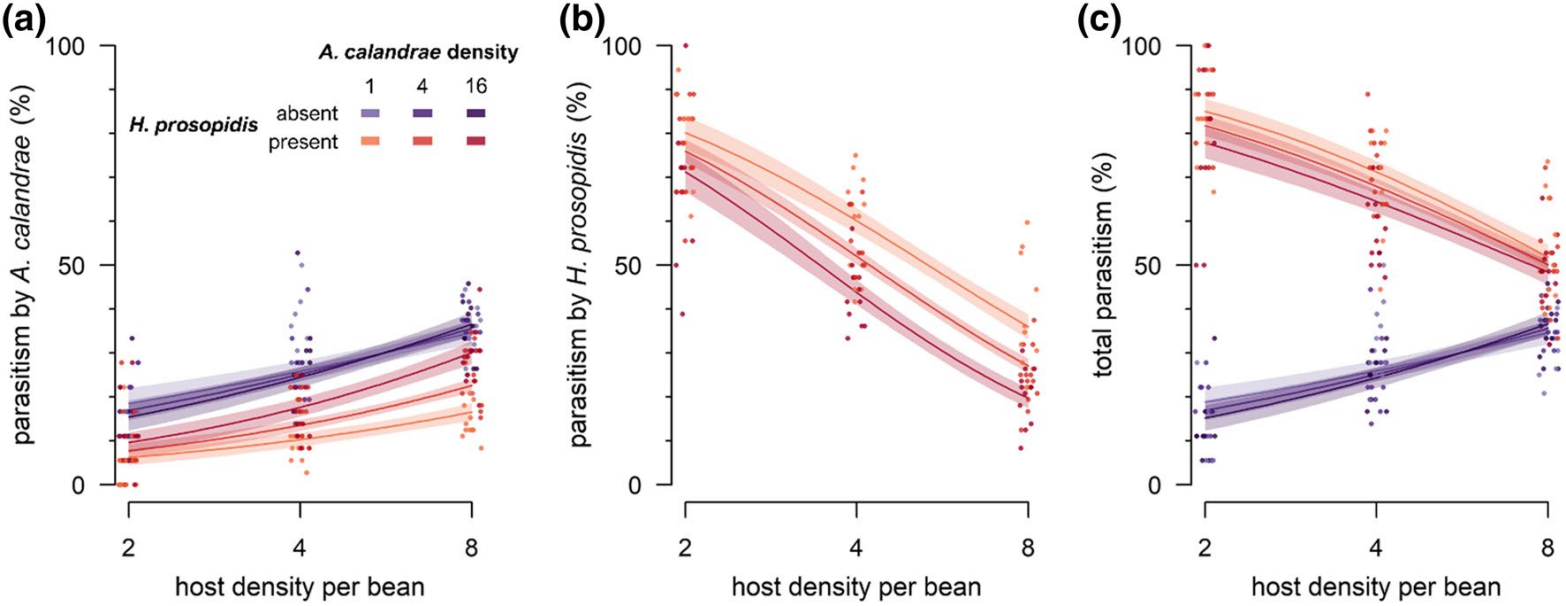
Percent parasitism by each or both parasitoids and its host density dependence. (**a**) Parasitism by the native parasitoid *Anisopteromalus calandrae*; (**b**) parasitism by the nonnative parasitoid *Heterospilus prosopidis*; (**c**) total parasitism. Purple: *H. prosopidis* absent; red: *H. prosopidis* present*.* Solid lines depict the logistic regression model fit (with shaded 95% CI). Jittering as in Fig. 1.

Since the model fit to the proportion of hosts parasitized by *A. calandrae* was not improved by a three-way interaction (*χ*^2^ = 1.8, *df* = 1, *p* > 0.1), this term was excluded from further analysis. The proportion parasitized by *A. calandrae* increased with increasing host and *A. calandrae* density but was decreased by the presence of *H. prosopidis* (Table 3, Fig. 3a). Additionally, it increased with increasing *A. calandrae* density only in the presence of *H. prosopidis* but was not affected by interactions between host density and *A. calandrae* density or between host density and the presence of *H. prosopidis* (Table 3, Fig. 3a). The proportion of hosts parasitized by *H. prosopidis* decreased at higher densities of hosts and at higher densities of *A. calandrae* but was not affected by an interaction between host density and *A. calandrae* density (Table 3, Fig. 3b).

**Table 3:** Effect tests for the logistic regression model of parasitism as a function of host density, the density of the native parasitoid *Anisopteromalus calandrae*, the presence/absence of the nonnative parasitoid *Heterospilus prosopidis*, and all two-way interactions.

Dependent variable	Independent variable	Likelihood ratio *χ*^2﻿^	*df*	*p*
Parasitism by *A. calandrae*	Log (host density)	219.0	1	< 0.001
	Log (*A. calandrae* density)	27.8	1	< 0.001
	Presence of *H. prosopidis*	185.2	1	< 0.001
	Log (host density) × log(*A. calandrae* density)	2.5	1	0.116
	Log (host density) × presence of *H. prosopidis*	2.3	1	0.127
	Log (*A. calandrae* density) × presence of *H. prosopidis*	30.5	1	< 0.001
Parasitism by *H. prosopidis*	Log (host density)	601.4	1	< 0.001
	Log (*A. calandrae* density)	83.8	1	< 0.001
	Log (host density) × log(*A. calandrae* density)	2.2	1	0.140
Parasitism by both parasitoids	Log (host density)	13.0	1	< 0.001
	Log (*A. calandrae* density)	4.7	1	0.030
	Presence of *H. prosopidis*	862.4	1	< 0.001
	Log (host density) × log(*A. calandrae* density)	4.2	1	0.041
	Log (host density) × presence of *H. prosopidis*	367.3	1	< 0.001
	Log (*A. calandrae* density) × presence of *H. prosopidis*	4.1	1	0.043

As the model fit to the proportion of hosts parasitized by both parasitoids was not improved by a three-way interaction (*χ*^2^ = 1.7, *df* = 1, *p* > 0.1), this term was excluded from the model. The proportion parasitized by both parasitoids decreased with increasing host and *A. calandrae* density and increased in the presence of *H. prosopidis* (Table 3, Fig. 3c). It was affected by interactions between host density and the presence of *H. prosopidis*, between host density and *A. calandrae* density, and between *A. calandrae* density and the presence of *H. prosopidis* (Table 3). For the interaction between host density and the presence of *H. prosopidis*, the proportion parasitized (by *A. calandrae*) increased with host density in the absence of *H. prosopidis*, but in the presence of *H prosopidis*, the total percent parasitism by both parasitoids decreased with host density (Fig. 3c). For the interaction between host density and *A. calandrae* density, the proportion parasitized decreased with increasing *A. calandrae* density at low host densities but was unaffected by *A. calandrae* density at high host densities. For the interaction between *A. calandrae* density and the presence of *H. prosopidis*, the proportion parasitized was unaffected by *A. calandrae* density in the absence of the nonnative parasitoid, but in the presence of the nonnative parasitoid, decreased with increasing *A. calandrae* density.

**Figure 4:**
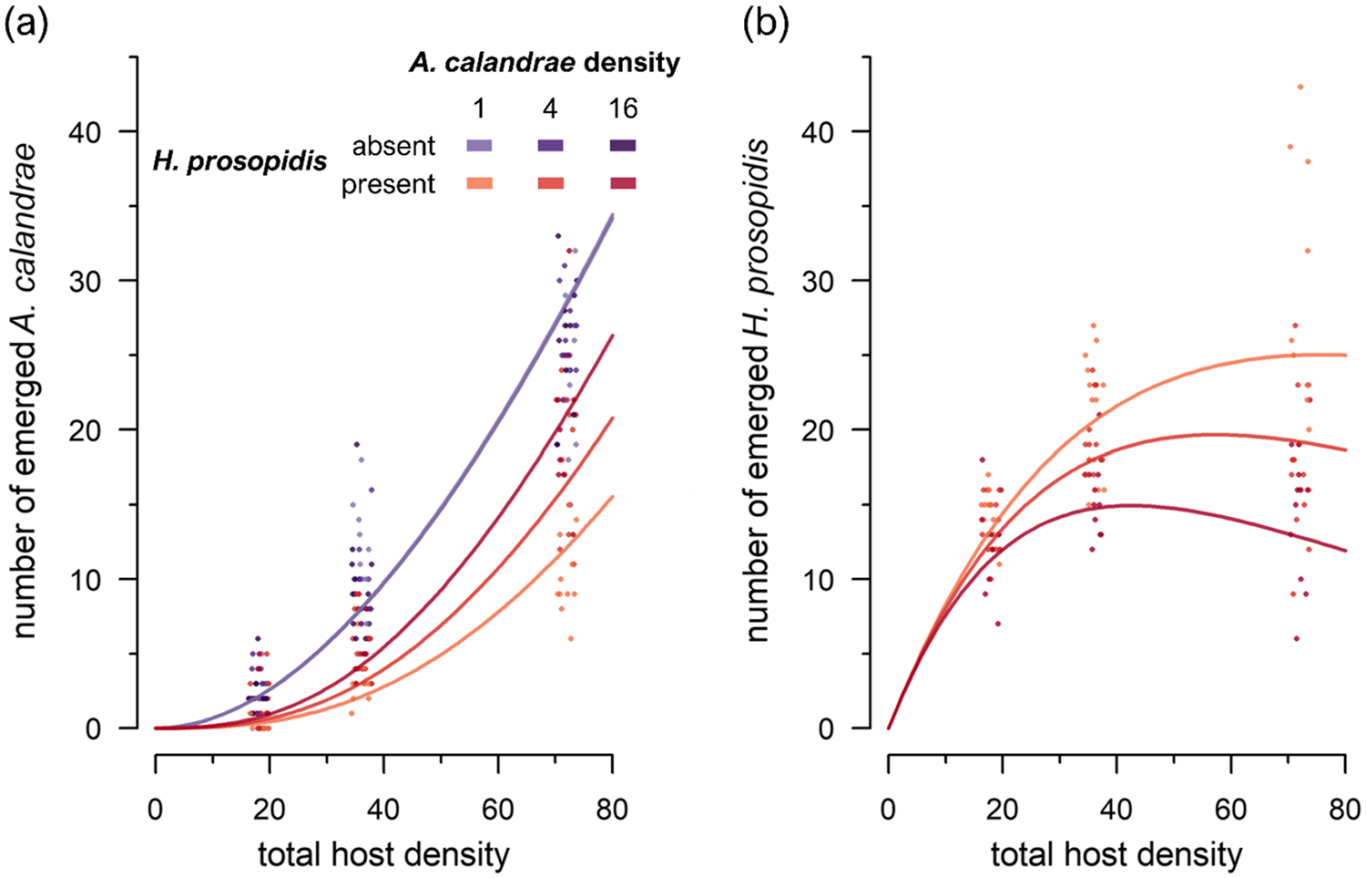
Population responses to host density for (**a**) the native parasitoid *Anisopteromalus calandrae* and (**b**) the nonnative parasitoid *Heterospilus prosopidis*. Solid lines depict the prediction by our Nicholson–Bailey-type model. Note that the model lines for *H. prosopidis* absent (purple) overlap considerably among different *A. calandrae* densities. Jittering as in Fig. 1.

Finally, the proportion parasitism data was fit to the Nicholson–Bailey-type model by optimizing the parameter values (*a*_*Ac*_ = 0.00703 ± 0.00055, *m* = 1.004 ± 0.044, *c*_*m*_ = 0.0441 ± 0.0082, *a*_*Hp*_ = 0.299 ± 0.044, *t*_*h*_ = 0.115 ± 0.017; estimate ± approximate SE) (Supplementary Table S1). The native parasitoid exhibited a Type I population response irrespective of its own density or of the presence/absence of the nonnative parasitoid (Fig. 4a). The population response of the nonnative parasitoid was a saturating Type II function when *A. calandrae* densities were low but switched to a dome-shaped Type IV function as *A. calandrae* density increased (Fig. 4b). We confirmed that further modifications of the model did not improve the model fit (Supplementary Table S1).

## Discussion

Mutual interference among the native parasitoid *A. calandrae* was reduced by the presence of the nonnative parasitoid *H. prosopidis* independent of host density, supporting our first hypothesis. Next, the proportion of female offspring produced by the native parasitoid was lower in the presence of the nonnative parasitoid, supporting our second hypothesis. Third, the contribution of the nonnative parasitoid to the total proportion of parasitized hosts was large at low host densities but decreased as host density increased, which was predicted by our third hypothesis. Finally, the Type II functional response of the nonnative parasitoid (which saturates as host density increases) shifted to a dome-shaped Type IV response (which decreases as host density increases past a certain threshold) as the density of the native parasitoid increased, leading us to reject our fourth hypothesis. Overall, our results at the population and community levels for two generations confirm previous work at the individual and community levels, which were studied for one generation and for 20 or more generations, respectively^6,24^. This indicates that the underlying mechanism of destabilized community dynamics induced by a nonnative parasitoid—the reduction of mutual interference among native parasitoids—is consistent across multiple levels of ecological organization as well as across different time scales. Additionally, our results regarding offspring sex ratio confirm predictions based on sex allocation theory^25,26,44^. Moreover, with the rejection of our fourth hypothesis, our results offer new insights into the effects of intra- and interspecific interactions between multiple parasitoids.

Intraspecific interference competition, or mutual interference, is a common antagonistic behavior among predators and parasitoids that reduces the searching efficiency of foraging individuals. While mutual interference stabilizes host–parasitoid (or prey–predator) systems, the presence of additional species can influence the degree of mutual interference via direct and indirect interactions^6,10,11,21^. As found in previous studies, the introduction of the nonnative parasitoid *H. prosopidis* leads to chaos for the otherwise oscillatory population dynamics of the azuki bean beetle and its native parasitoid *A. calandrae*^6^. The high searching efficiency and lack of self-regulation of the nonnative parasitoid reduce the already low host densities to the point that there are a limited number of hosts available to the native parasitoid for host feeding; this leads to synergistic limitations in egg maturation and a subsequent reduction in fecundity^6,41^ (which is also one of the underlying mechanisms in our model). This lowers the reproductive activity of the native parasitoid, reducing the magnitude of mutual interference. At the individual level, the presence of the nonnative parasitoid reduces the number of antagonistic intraspecific interactions between native parasitoids and decreases their host-feeding behavior, thus reducing overall searching efficiency, at a low host density^24^. Adding to these prior works, our experimental results provide population-level evidence that the nonnative parasitoid reduces mutual interference among the native parasitoid independent of host density.

The offspring female ratio of *A. calandrae* was unaffected by conspecific density but did decrease in the presence of the nonnative parasitoid. This first finding was expected because solitary parasitoids such as *A. calandrae* are less likely to experience local mate competition than gregarious parasitoids^44,46,48^ (but see^27^). The latter finding supports the prediction that mothers in a favorable environment (e.g. one with abundant resources) will invest more in the offspring sex that will benefit more from that environment^25,26^; for solitary, synovigenic parasitoids such as *A. calandrae*, an environment with ample hosts for host feeding is more beneficial for female offspring than for male offspring because females can utilize these resources to increase their lifetime reproductive success (whereas males do not host feed). However, when competing with the parasitoid *Choetospila elegans* (Hymenoptera: Pteromalidae) for rice weevils, the offspring sex ratio of *A. calandrae* was not affected by interspecific competition, likely because of its relative dominance as a competitor^49,50^. In our system, the nonnative parasitoid *H. prosopidis* is the superior competitor because of its higher searching efficiency and priority effects^6^ (when both host and *A. calandrae* densities are low). This is typical of specialists with relatively narrow host ranges. Generalist parasitoids, such as *A. calandrae*, can modify the sex ratio of their offspring more easily^51^. Here, the effects of host stage should also be considered because unparasitized larvae develop into pupae during the 4-day exposure to the parasitoids^28,52^. When in competition, the parasitization preference of *A. calandrae* shifts toward pupae, whereas that of the nonnative parasitoid shifts toward fourth instar larvae (niche shift^28^), leaving a greater proportion of suitable hosts (pupae) for *A. calandrae*. This proportional increase in suitable hosts is predicted to increase the proportion of female offspring produced by *A. calandrae* in the absence of the effects of host depletion^27^. Our experiment showed that the proportion of female offspring produced by *A. calandrae* actually decreased in the presence of the nonnative parasitoid. This indicates that host depletion—rather than niche shift—affected sex allocation by the native parasitoid, which supports the existing sex allocation theory^25^.

Whereas the presence of the nonnative parasitoid greatly increased the overall level of parasitism at low host densities, the contribution of the nonnative parasitoid to the level of parasitism at higher host densities was small. When multiparasitism by the two parasitoids occurs (in which both parasitoid species parasitize the same host individual), the probability of winning direct interspecific competition is nearly 0.5 for the larvae of both species^53^. However, frequent host feeding by *A. calandrae* at higher host densities, even in the presence of *H. prosopidis*^24^, could reduce host availability for *H. prosopidis* and lead to the reversal of competitive dominance. Analyses based on assemblage-level dynamics likely capture the population responses of each species at typical densities (possibly near equilibrium), and we might miss out on global dynamical mechanisms away from such ecological states (especially when correlations between biological parameters are high). Nevertheless, the efficacy of the native parasitoid alone, combined with its increasingly female-biased sex ratio at higher host densities, has important implications for the paradigm of single vs. multiple introductions in biological control^20^ (also see^54^). With the additional context provided by the present study, this suggests that, when the nonnative parasitoid is present, a related reduction in the searching efficiency of the native parasitoid via decreased host feeding likely leads to an even greater decrease in its fecundity (and potentially longevity^55^) that would further destabilize the dynamics of the system^6,56^.

As our study demonstrates, indirect competition over a shared resource can also bring about changes in sex allocation (reducing the proportion of female offspring), which, in turn, may have important consequences for the population dynamics of the system, such as higher host equilibrium densities and decreased stability^57,58^. Likewise, the effects of interspecific interactions on mutual interference behavior can also affect the population and community levels of organization, since the higher-order effects of mutual interference range from influencing predator functional response^59,60^ to affecting patterns in species distribution, persistence, and stability^11,61–63^.

While mutual interference can accommodate the coexistence of multiple consumers under certain conditions^64^, emergent effects have important consequences for population- and community-level stability and persistence. Furthermore, as mutual interference results in a predator-dependent rate of prey consumption, it is related to the availability and variability of resources across space^63,65,66^. Our current study bridges the gap between predictions based on population models and the short-term and small-scale properties of behavioral interactions observed in a multispecies assemblage. Ongoing global processes, including climate change (which alters the distribution and phenology of species with different reproductive strategies^67–69^) as well as the spread of nonnative or invasive species, will likely change the composition, function, traits, and stability of multispecies systems. The present study is just one example of how direct and indirect competitive interactions can influence the population dynamics of consumer–resource interactions^70,71^.

## Supplementary Information

Supplementary Information.

## Data Availability

Data will be provided upon reasonable request.

## References

[1] Sih A, Crowley P, McPeek M, Petranka J, Strohmeier K (1985). Predation, competition, and prey communities: A review of field experiments. Annu. Rev. Ecol. S..

[2] Schmitz OJ, Grabowski JH, Peckarsky BL, Preisser EL, Trussell GC, Vonesh JR (2008). From individuals to ecosystem function: Toward an integration of evolutionary and ecosystem ecology. Ecology.

[3] Sih A, Englund G, Wooster D (1998). Emergent impacts of multiple predators on prey. Trends Ecol. Evol..

[4] Holt RD (1977). Predation, apparent competition, and structure of prey communities. Theor. Popul. Biol..

[5] Bonsall MB, Hassell MP (1997). Apparent competition structures ecological assemblages. Nature.

[6] Tuda M, Shimada M (2005). Complexity, evolution, and persistence in host–parasitoid experimental systems with *Callosobruchus* beetles as the host. Adv. Ecol. Res..

[7] Briggs CJ, Nisbet RM, Murdoch WW (1993). Coexistence of competing parasitoid species on a host with a variable life cycle. Theor. Popul. Biol..

[8] Peri E, Cusumano A, Amodeo V, Wajnberg E, Colazza S (2014). Intraguild interactions between two egg parasitoids of a true bug in semi-field and field conditions. PLoS ONE.

[9] Pekas A, Tena A, Harvey JA, Garcia-Marí F, Frago E (2016). Host size and spatiotemporal patterns mediate the coexistence of specialist parasitoids. Ecology.

[10] DeLong JP, Vasseur DA (2011). Mutual interference is common and mostly intermediate in magnitude. BMC Ecol..

[11] Hassell MP, Varley GC (1969). New inductive population model for insect parasites and its bearing on biological control. Nature.

[12] Hassell MP (1971). Mutual interference between searching insect parasites. J. Anim. Ecol..

[13] Charnov EL, Orians GH, Hyatt K (1976). Ecological implications of resource depression. Am. Nat..

[14] Free CA, Beddington JR, Lawton JH (1977). On the inadequacy of simple models of mutual interference for parasitism and predation. J. Anim. Ecol..

[15] Visser ME, Jones TH, Driessen G (1999). Interference among insect parasitoids: A multi-patch experiment. J. Anim. Ecol..

[16] Beddington JR (1975). Mutual interference between parasites or predators and its effect on searching efficiency. J. Anim. Ecol..

[17] DeAngelis DL, Goldstein RA, O’Neill RV (1975). A model for trophic interaction. Ecology.

[18] Arditi R, Callois JM, Tyutyunov Y, Jost C (2004). Does mutual interference always stability predator–prey dynamics? A comparison of models. C. R. Biol..

[19] Abrams PA (2015). Why ratio dependence is (still) a bad model of predation. Biol. Rev..

[20] Pedersen BS, Mills NJ (2004). Single vs. multiple introduction in biological control: The roles of parasitoid efficiency, antagonism, and niche overlap. J. Appl. Ecol..

[21] Amarasekare P (2002). Interference competition and species coexistence. Proc. R. Soc. B.

[22] Mohamad R, Wajnberg E, Monge JP, Goubault M (2015). The effect of direct interspecific competition on patch exploitation strategies in parasitoid wasps. Oecologia.

[23] Elliott JM (2004). Interspecific interference and the functional response of four species of carnivorous stoneflies. Freshw. Biol..

[24] Nakamichi Y, Tuda M, Wajnberg E (2020). Intraspecific interference between native parasitoids modified by a non-native parasitoid and its consequence on population dynamics. Ecol. Entomol..

[25] Trivers RL, Willard DE (1973). Natural selection of parental ability to vary the sex ratio of offspring. Science.

[26] Appleby BM, Petty SJ, Blakey JK, Rainey P, Macdonald DW (1997). Does variation of sex ratio enhance reproductive success of offspring in tawny owls (*Strix aluco*)?. Proc. R. Soc. B.

[27] Nishimura K, Jahn GC (1996). Sex allocation of three solitary ectoparasitic wasp species on bean weevil larvae: Sex ratio change with host quality and local mate competition. J. Ethol..

[28] Shimada M, Fujii K (1985). Niche modification and stability of competitive systems. I. Niche modification process. Res. Popul. Ecol..

[29] Utida S (1957). Population fluctuation, an experimental and theoretical approach. Cold Spring Harb. Symp. Quant. Biol..

[30] Utida S (1957). Cyclic fluctuations of population density intrinsic to the host–parasitoid system. Ecology.

[31] Fujii K (1968). Studies on the interspecies competition between the azuki bean weevil and the southern cowpea weevil. III. Some characteristics of strains of two species. Res. Popul. Ecol..

[32] Bellows TS (1982). Analytical models for laboratory populations of *Callosobruchus chinensis* and *C. maculatus* (Coleoptera, Bruchidae). J. Anim. Ecol..

[33] Tuda M (1993). Density dependence depends on scale; at larval resource patch and at whole population. Res. Popul. Ecol..

[34] Tuda M, Shimada M (1995). Developmental schedules and persistence of experimental host–parasitoid systems at two different temperatures. Oecologia.

[35] Tuda M, Chou L-Y, Niyomdham C, Buranapanichpan S, Tateishi Y (2005). Ecological factors associated with pest status in *Callosobruchus* (Coleoptera: Bruchidae): High host specificity of non-pests to Cajaninae (Fabaceae). J. Stored Prod. Res..

[36] Tuda M, Rönn J, Buranapanichpan S, Wasano N, Arnqvist G (2006). Evolutionary diversification of the bean beetle genus *Callosobruchus* (Coleoptera: Bruchidae): Traits associated with stored-product pest status. Mol. Ecol..

[37] Tuda M (2007). Applied evolutionary ecology of insects in the subfamily Bruchinae (Coleoptera: Chrysomelidae). Appl. Entomol. Zool..

[38] Clausen CP (1978). Introduced Parasites and Predators of Arthropod Pests and Weeds: A World Review.

[39] Schmale I, Wäckers FL, Cardona C, Dorn S (2001). Control potential of three hymenopteran parasitoid species against the bean weevil in stored beans: The effect of adult parasitoid nutrition on longevity and progeny production. Biol. Control.

[40] Vamosi SM, den Hollander MD, Tuda M (2011). Egg dispersion is more important than competition type for herbivores attacked by a parasitoid. Popul. Ecol..

[41] Shimada M (1999). Population fluctuation and persistence of one-host–two parasitoid systems depending on resource distribution: From parasitizing behavior to population dynamics. Res. Popul. Ecol..

[42] Baker JE, Perez-Mendoza J, Beeman RW (1998). Multiple mating potential in a pteromalid wasp determined by using an insecticide resistance marker. J. Entomol. Sci..

[43] Yamamura K (1999). Transformation using (x + 0.5) to stabilize the variance of populations. Popul. Ecol..

[44] Hamilton WD (1967). Extraordinary sex ratios. Science.

[45] Waage JK, Lane JB (1984). The reproductive strategy of a parasitic wasp: II. Sex allocation and local mate competition in *Trichogramma evanescens*. J. Anim. Behav..

[46] Strand MR (1988). Variable sex ratio strategy of *Telonomus heliothidis* (Hymenoptera: Scelionidae): Adaptation to host and conspecific density. Oecologia.

[47] Hassell MP (1978). The Dynamics of Arthropod Predator-Prey Systems.

[48] Godfray HCJ (1994). Parasitoids: Behavioral and Evolutionary Ecology.

[49] Wen B, Smith L, Brower JH (1994). Competition between *Anisopteromalus calandrae* and *Choetospila elegans* (Hymenoptera: Pteromalidae) at different parasitoid densities on immature maize weevils (Coleoptera: Curculionidae) in corn. Environ. Entomol..

[50] Wen B, Brower JH (1995). Competition between *Anisopteromalus calandrae* and *Choetospila elegans* (Hymenoptera: Pteromalidae) at different parasitoid densities on immature rice weevils (Coleoptera: Curculionidae) in wheat. Biol. Control.

[51] Campan E, Benrey B (2004). Behavior and performance of a specialist and a generalist parasitoid of bruchids on wild and cultivated beans. Biol. Control.

[52] Choi WI, Yoon TJ, Ryoo MI (2001). Host-size-dependent feeding behaviour and progeny sex ratio of *Anisopteromalus calandrae* (Hym., Pteromalidae). J. Appl. Entomol..

[53] Wai, K. M. Intra- and interspecific larval competition among wasps parasitic to bean weevil larvae. Thesis—University of Tsukuba, D.Sc. (A), no. 714 (1990).

[54] Heimpel GE, Cock MJW (2018). Shifting paradigms in the history of classical biological control. Biocontrol.

[55] Miksanek JR, Heimpel GE (2020). Density-dependent lifespan and estimation of life expectancy for a parasitoid with implications for population dynamics. Oecologia.

[56] Kidd NAC, Jervis MA (1989). The effects of host-feeding behaviour on the dynamics of parasitoid–host interactions, and the implications for biological control. Res. Popul. Ecol..

[57] Comins HN, Wellings PW (1985). Density-related parasitoid sex-ratio: Influence on host–parasitoid population dynamics. J. Anim. Ecol..

[58] Hassell MP, Waage JK, May RM (1983). Variable parasitoid sex ratios and their effect on host–parasitoid dynamics. J. Anim. Ecol..

[59] Skalski GT, Gilliam JF (2001). Functional responses with predator interference: Viable alternatives to the Holling Type II model. Ecology.

[60] Kratina P, Vos M, Bateman A, Anholt BR (2008). Functional responses modified by predator density. Oecologia.

[61] Freedman HI (1979). Stability analysis of a predator–prey system with mutual interference and density-dependent death rates. Bull. Math. Biol..

[62] Erbe LH, Freedman HI (1985). Modeling persistence and mutual interference among subpopulations of ecological communities. Bull. Math. Biol..

[63] Alonso D, Bartumeus F, Catalan J (2002). Mutual interference between predators can give rise to Turing spatial patterns. Ecology.

[64] May RM, Hassell MP (1981). The dynamics of multiparasitoid–host interactions. Am. Nat..

[65] Wajnberg E, Curty C, Colazza S (2004). Genetic variation in the mechanisms of direct mutual interference in a parasitic wasp: Consequences in terms of patch-time allocation. J. Anim. Ecol..

[66] Okuyama T (2016). Parasitoid aggregation and interference in host–parasitoid dynamics. Ecol. Entomol..

[67] Jeffs CT, Lewis OT (2013). Effects of climate warming on host–parasitoid interactions. Ecol. Entomol..

[68] Laws AN (2017). Climate change effects on predator–prey interactions. Curr. Opin. Insect Sci..

[69] Tougeron K, Brodeur J, Le Lann C, van Baaren J (2020). How climate change affects the seasonal ecology of insect parasitoids. Ecol. Entomol..

[70] Tuda M, Bonsall MB (1999). Evolutionary and population dynamics of host–parasitoid interactions. Res. Popul. Ecol..

[71] Outreman Y, Andrade TO, Louâpre P, Krespi L, Violle C, van Baaren J (2018). Multi-scale and antagonist selection on life-history traits in parasitoids: A community ecology perspective. Funct. Ecol..

