# Supplementary material for: Impact of a nonnative parasitoid species on intraspecific interference and offspring sex ratio
Source: Sci Rep. 2021 Dec 1;11:23215. doi: 10.1038/s41598-021-02713-1 (PMC8636619; doi:10.1038/s41598-021-02713-1)
Supplement: Supplementary file 1 — Supplementary Information. [file 41598_2021_2713_MOESM1_ESM.docx]

**Impact of a nonnative parasitoid species on intraspecific interference and offspring sex ratio**

Yao Zhuo Zhang^1^, Zhengya Jin^2^, James Rudolph Miksanek^3^, Midori Tuda^4*^

^1^Laboratory of Insect Natural Enemies, Graduate School of Bioresource and Bioenvironmental Sciences, Kyushu University, Fukuoka 819-0395, Japan

^2^ Guangdong Key Laboratory for Innovation Development and Utilization of Forest Plant Germplasm, College of Forestry and Landscape Architecture, South China Agricultural University, Guangzhou 510642, China

^3^Department of Entomology, University of Minnesota, Saint Paul, Minnesota 55108, United States

^4^Laboratory of Insect Natural Enemies, Institute of Biological Control, Faculty of Agriculture, Kyushu University, Fukuoka 819-0395, Japan

^*^Correspondence:

Tuda, M.

**Supplementary Table S1.** Model selection. None of the modifications on the default model (Eqs. 2–5) decreased the corrected AIC (AICc) score, indicating that the default model was the best choice.

| Parameter modified | Definition | Number of parameters | AICc |
| --- | --- | --- | --- |
| None [default model (Eqs. 2–5)] | *A. calandrae*: host-feeding, Type I functional response, and mutual interference affected by *H. prosopidis*.  *H. prosopidis*: Type II functional response | 5 | −717.6 |
| *c_m_*= 0 | No effect of *H. prosopidis* on mutual interference among *A. calandrae*. | 4 | −686.3 |
| *m* = 0 | No mutual interference among *A. calandrae*. | 3 | −536.2 |
| No *H*_0_ in Eq. 4 | No host feeding by *A. calandrae*. | 5 | −636.4 |
| *t_h_* = 0 | Type I functional response for *H. prosopidis.* | 4 | −648.9 |
